# Polygalacic acid attenuates cognitive impairment by regulating inflammation through PPARγ/NF‐κB signaling pathway

**DOI:** 10.1111/cns.14581

**Published:** 2024-02-08

**Authors:** Tan Zhao, Jianping Jia

**Affiliations:** ^1^ Innovation Center for Neurological Disorders and Department of Neurology, Xuanwu Hospital Capital Medical University, National Clinical Research Center for Geriatric Diseases Beijing China; ^2^ Beijing Key Laboratory of Geriatric Cognitive Disorders Beijing China; ^3^ Clinical Center for Neurodegenerative Disease and Memory Impairment Capital Medical University Beijing China; ^4^ Center of Alzheimer's Disease Beijing Institute of Brain Disorders, Collaborative Innovation Center for Brain Disorders, Capital Medical University Beijing China; ^5^ Key Laboratory of Neurodegenerative Diseases, Ministry of Education Beijing China

**Keywords:** Alzheimer's disease, apoptosis, inflammation, Polygalacic acid

## Abstract

**Aims:**

We aimed to explore the role and molecular mechanism of polygalacic acid (PA) extracted from traditional Chinese medicine Polygala tenuifolia in the treatment of Alzheimer's disease (AD).

**Methods:**

The network pharmacology analysis was used to predict the potential targets and pathways of PA. Molecular docking was applied to analyze the combination between PA and core targets. Aβ42 oligomer‐induced AD mice model and microglia were used to detect the effect of PA on the release of pro‐inflammatory mediators and its further mechanism. In addition, a co‐culture system of microglia and neuronal cells was constructed to assess the effect of PA on activating microglia‐mediated neuronal apoptosis.

**Results:**

We predict that PA might regulate inflammation by targeting PPARγ‐mediated pathways by using network pharmacology. In vivo study, PA could attenuate cognitive deficits and inhibit the expression levels of inflammation‐related factors. In vitro study, PA can also decrease the production of activated microglia‐mediated inflammatory cytokines and reduce the apoptosis of N2a neuronal cells. PPARγ inhibitor GW9662 inversed the neuroprotective effect of PA. Both in vivo and in vitro studies showed PA might attenuate the inflammation through the PPARγ/NF‐κB pathway.

**Conclusions:**

PA is expected to provide a valuable candidate for new drug development for AD in the future.

## Graphical abstract

Polygalacic acid can decrease the production of activated microglia‐mediated inflammatory cytokines and reduce the apoptosis of N2a neuronal cells through PPARγ/NF‐κB pathway.

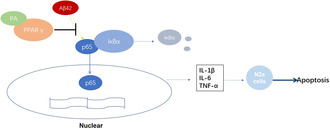

## 1 INTRODUCTION

Approximately, 47 million people worldwide are living with dementia of which most are affected with Alzheimer's disease (AD), a devastating form of a neurodegenerative disorder.^1^ Cognitive decline, commonly first recognized as memory impairment, amyloid β (Aβ) plaques, and neurofibrillary tangles (NFT) are typical clinical features and core pathologies of AD.^2^, ^3^ Studies showed that patients may exhibit Aβ plaque pathology for up to or greater than a decade before any overt diagnosis of AD.^4^ Trials aiming to reduce the production and burden of Aβ aggregation within the brain are still underway,^5^ although the United States Food and Drug Administration approved lecanemab and aducanumab for treatment of AD.^6^ Previous clinical studies showed a gap between the Aβ pathology and disease progression and suggested that other pathological mechanisms may be driving both the onset of the disorder and the progression of the disease.^7^

Increasing evidence indicates that sustained inflammation mediated by neuroglial cells is the main cause of cognitive impairment and neurodegenerative disease progression in AD patients.^8^, ^9^, ^10^ The presence of inflammatory markers was found in the brains of patients, including elevated levels of cytokines in serum along with microgliosis.^11^, ^12^ The increase in these molecules is positively correlated to cognitive impairment at different stages of AD as well as in individuals with mild cognitive impairment.^13^ Studies have also demonstrated that Aβ aggregation‐related neuroinflammation causes neuronal damage and clinical deterioration.^14^ In the early stages of AD, activated microglia can phagocytose and degrade Aβ, which has a protective effect. However, activated microglia also release a large number of inflammatory cytokines, such as interleukin‐1β (IL‐1β), interleukin‐6 (IL‐6), interleukin‐8 (IL‐8), and tumor necrosis factor‐α (TNF‐α), which can further lead to neuronal damage and death.^15^ Therefore, targeting the inflammation is one potential strategy to treat AD.

Some natural products present promising effects on AD.^16^ Experimental evidence demonstrates anti‐inflammatory effects and decreased Aβ levels induced by natural compounds.^17^, ^18^ Polygalacic acid (PA) is extracted from the root of Polygala tenuifolia, which is a traditional Chinese herbal medicine and has been used for the treatment of AD for a long history.^19^ Previous studies showed that chemical components extracted from Polygala tenuifolia have significant therapeutic effects in the treatment of AD through anti‐inflammatory pathways.^17^, ^19^ Saponins are the main active ingredients in the extracted components, and most of the saponins are unstable and can be hydrolyzed to generate PA.^20^, ^21^ However, the research on PA and AD are lacking. Therefore, in this study, we mainly investigate the pharmacological roles of PA in the treatment of AD.

Network pharmacology is a promising way to reveal the relationship between small molecules, therapeutic targets, and diseases. With the aid of a biological network, it is convenient to explore the potential targets and mechanisms of pharmacological ingredients.^22^ Based on previous studies, we assumed that PA can protect cognitive function by modulating inflammation in AD. To verify our hypothesis, we first constructed an Aβ‐oligomer‐induced mice model to investigate whether PA could improve cognitive impairment in vivo. Then we utilized network pharmacology analysis to predict potential core targets and signaling pathways of PA. Subsequently, we constructed Aβ oligomers‐stimulated BV2 microglia and N2a neuron cells to further explore the mechanisms of PA by adding inhibitors of core targets.

## 2 MATERIALS AND METHODS

### 2.1 Drugs and reagents

Polygalacic acid (Analytical standard, HPLC ≥98%) was purchased from PUSH Bio‐Technology Co., Ltd (Chengdu, China). Aβ42 oligomer powder was obtained from ChinaPeptides Co., Ltd. (Suzhou, China). Enzyme‐linked immunosorbent assay (ELISA) was purchased from Cusabio Co. LTD (Wuhan, China). Phosphate‐buffered saline (PBS) was obtained from Hyclone (Logan, UT, USA). Dimethyl sulfoxide (DMSO) and 3‐(4,5‐dimethyl‐2‐thiazolyl)‐2,5‐diphenyl‐2 H‐tetrazolium bromide (MTT) were supplied by Sigma‐Aldrich (Missouri, USA). Blocking goat serum, Triton X‐100, DAPI nuclear staining, and antiquenching sealer for immunofluorescence analysis were purchased from Solarbio (Beijing, China). Dulbecco's modified Eagle's medium (DMEM) and fetal bovine serum (FBS) were purchased from Gibco (NY, USA). The BCA Protein Assay Kit and annexin V‐FITC/PI apoptosis detection kit were purchased from Beyotime Biotechnology Co., Ltd. (Shanghai, China). Antibodies against NF‐κB p65, p‐NF‐κB p65, Iκβα, p‐Iκβα, and Histone H3 were purchased from Cell Signaling Technology (MA, USA). PPARγ and GAPDH were bought from Proteintech (Wuhan, China). Iba‐1 antibody was bought from Thermo Fisher (Waltham, USA). NeuN antibodies and secondary antibodies for western blotting and immunohistochemistry were purchased from Abcam (Cambridge, UK) and Proteintech (Wuhan, China).

### 2.2 Animals and experiment design

Thirty‐eight 2‐month‐old wild‐type C57BL‐6 male mice, weighing between 18 and 22 g, were purchased from Viewsolid Biotech (Beijing, China). They were maintained under controlled conditions of a temperature of 22 ± 1°C with a relative humidity of 40%–60% and a regular 12 h light/dark cycle. All mice acclimatized for at least 1 week and had free access to water and food. All procedures were approved by the Institutional Animal Ethics Committee, Capital Medical University.

Preparation of Aβ42 oligomers was based on previous methods.^23^ The human Aβ42 peptide was reconstituted and dissolved in phosphate‐buffered solution at a concentration of 410 pmol/5 μL. Mice were weighed and anesthetized by pentobarbital sodium solution (40 mg/kg, intraperitoneally injected) before the lateral intracerebroventricular injection (i.c.v.). Aβ42 (5 μL/5 min/mouse) or phosphate buffered solution was injected at a rate of 1 μL/5 min into the mice ventricles, by using a Hamilton micro‐syringe in a coordinate of 0.5 mm anteroposterior (AP), 1.5 mm dorsoventral (DV), and 1.5 mm mediolateral (ML) to the bregma. The injector was left in the injection site for 5 min.^24^ The spatial memory deficits lasted for more than 3 weeks after the i.c.v. administration of Aβ42.^25^ The mice were randomly divided as wild‐type control, Aβ42‐alone‐treated group, Aβ42 + low dose (6 mg/kg/day), and Aβ42 + high dose of PA‐treated group (12 mg/kg/day for 3 weeks, dissolved in 0.5% sodium carboxymethylcellulose) with eight mice per group.^21^ The PA groups were administered orally once per day for 3 weeks, while mice in control and Aβ42‐ alone treated group received an equal volume of 0.5% sodium carboxymethylcellulose. Behavioral tests were initiated on the 14 days of drug administration and lasted for 1 week. On the last day of the animal experiment, mice were transcardially perfused with PBS and the brains were harvested. One‐half of the brain was immersed in 4% paraformaldehyde, followed by xylene treatment and embedding in paraffin. The remaining brain tissues were immediately stored at −80°C for biochemical analyses.

#### 2.2.1 Y‐maze test

The Y‐maze can be used to assess short‐term memory in mice.^26^ The Y‐maze is a device composed of three arms (35 cm long, 5 cm wide, and 15 cm above the ground) which connect with each other at a 120° angle. During the test, the mice were introduced at the distal end of one arm and allowed to explore the arms freely for 8 min. The order and the number of mice entering arms were recorded. An entry occurs when all four limbs of the mouse are within an arm. One alternation behavior was defined as consecutive entries into all three arms. The alternation behavior rate was calculated as alternation behavior (%) = number of alternations/(number of arm entries − 2) × 100%.

#### 2.2.2 Morris water maze test

The experimental procedure was based on the previous protocol.^27^ The Morris water maze consists of a stainless‐steel circular pool with a diameter of 100 cm and a height of 60 cm. The pool has a white background and is surrounded by silver‐gray curtains with four black and white patterns (star, triangle, square, and circle). The pool is divided into four virtual quadrants, each corresponding to one of four starting points (E, W, N, S). The water temperature is controlled at around 20 ± 2°C, and the room temperature is controlled at around 22 ± 1°C.

There are two phases of the Morris water maze test: the learning phase and the spatial probe phase. The learning phase reflects the acquisition and formation of memory and the spatial exploration experiment reflects the mice's memory retrieval ability. The learning phase lasted for 5 days, and the mice were trained in four trials each day. The maximum time for each trial was set to 60 s. If the mice find the platform within 60‐s limit, the software will record and automatically stop the experiment after 5 s, otherwise, the mice will be guided to the platform for an additional 15 s to reinforce the memory. After each trial, the mice were dried with a towel and placed back in their cages to rest for at least 20 min before the next trial. Two indices will be recorded in this phase: distance to target (cm) before climbing onto the platform, indicating the distance the mouse moved before successfully climbing onto the platform for the first time, and escape latency to target (s), indicating the time it takes for the mouse to find the platform. Successful climbing onto the platform is defined as the mouse staying on the platform for more than 5 s.

During the spatial probe phase (the sixth day), the escape platform was removed from the water, and each mouse was allowed to explore the water maze freely for 60 s. Time in the target quadrant(s) (the time spent in the quadrant where the platform used to be) and number of platform crosses (the number of crossings where the original platform used to be) were recorded to evaluate the spatial memory ability of the mice. The animals were video‐tracked using SMART3 software (Panlab HARVARD, USA).

### 2.3 Network pharmacology analysis

#### 2.3.1 Target prediction

We obtained the chemical structure of Polygalacic acid from PubChem (https://pubchem.ncbi.nlm.nih.gov/) and predicted the potential targets of Polygalacic acid through SwissTargetPrediction (http://swisstargetprediction.ch/) and SEA (https://sea.bkslab.org/), with “MaxTC” >0.3. Information on AD‐associated target genes was collected from Online Mendelian Inheritance in Man (OMIM, https://omim.org/) and GeneCards database (https://www.genecards.org/). Then the genes were normalized by the Uniport database and then sequenced by “Relevance score.” Drug targets and disease targets were mapped with Venny 2.1.0 (https://bioinfogp.cnb.csic.es/tools/venny/).

#### 2.3.2 Protein–protein interaction

The matched targets between PA and AD were selected for further construction of protein–protein interaction (PPI) network. These drug‐disease intersection genes were uploaded to the STRING database (https://cn.string‐db.org/) in this step. The interaction networks were imported into Cytoscape 3.8.2 for topology analysis. The key‐related targets of PA were screened based on the condition of node size, edge thickness, degree, closeness, and betweenness.

#### 2.3.3 Pathway enrichment analysis

Gene Ontology (GO) biological process (BP) and Kyoto Encyclopedia of Genes and Genomes (KEGG) enrichment analyses were performed with the DAVID database (https://david.ncifcrf.gov/). The pathways with *p* value ≤0.05 were selected in GO enrichment analysis and KEGG biological pathway enrichment, and the top 10 pathways including BP, molecular function (MF), and cellular component were obtained. The top 20 pathways related to AD were selected in the KEGG analysis. The pathways were visualized in bubble charts and histograms by using a bioinformatics platform (http://www.bioinformatics.com.cn/).

#### 2.3.4 Molecular docking

Molecular docking was performed to identify the binding mode of the PA toward proteins. The docking was carried out by AutoDock Vina (1.1.2) with key targets of PA and validated their interaction activity. The specific steps included the following: (1) download the compound in mol2 format from the TCMSP official website, perform energy minimization in Chembio3D, then add hydrogen, calculate charges, assign charges, and set rotatable bonds in AutoDockTools‐1.5.6; (2) the structures of proteins were extracted from Protein Data Bank (PDB), removed the original ligand and water molecules in PyMoL (2.3.0), then added hydrogen, calculate charges, assign charges, specify atomic types through AutoDocktools (v1.5.6); (3) set the protein's original ligand as the docking box center, or if there is no original ligand, set the docking area near the reported key amino acid residues, and set the grid box size to 50 × 50 × 50; and (4) finally, used PyMOL and Ligplot for interaction mode analysis.

### 2.4 Cell culture and treatments

Murine microglial cells BV2 and neuron cells N2a were obtained from the National Cell Line Resource Infrastructure (Beijing, China). Cells were cultured in a DMEM complete medium containing 10% FBS as well as 1% penicillin–streptomycin (Sigma‐Aldrich, St. Louis, MO, USA) in a humidified atmosphere with 37°C and 5% CO_2_.

#### 2.4.1 Cell viability assay

Cell viability was evaluated by the MTT assay. PA and Aβ42 oligomers power was dissolved in DMSO and prepared in different concentrations by the complete medium at the time of use. BV2 (8 × 10^3^ cells per well) and N2a cells (1 × 10^4^ cells per well) were cultured in 96‐well plates. The cells were then exposed to 24 h of PA or Aβ42 treatment at different concentrations to examine the neurotoxicity of PA and Aβ42 oligomers. The cells were incubated with PA for 2 h and then co‐incubated with Aβ42 oligomers for 24 h to evaluate the protective effect of PA against the neurotoxicity of Aβ42 oligomers. Four experimental groups were used: (1) control group, (2) Aβ42‐induced BV2 microglia, (3) Aβ42‐induced BV2 microglia with PA pretreated, and (4) cells pretreated with PPARγ inhibitor GW9662 (MedChemExpress, NJ, USA).

The MTT assay was also used to measure indirect toxicity under conditioned media. The BV2 cells were pretreated with PA for 2 h then incubated with Aβ42 oligomers for 10 h or pretreated with GW9662 2 h, then incubated with PA 2 h, and Aβ42 oligomers for 10 h. After that, a fresh medium was used to replace the supernatant of BV2 cells to culture for an additional 12 h. The supernatant of BV2 was collected as a conditioned medium and was added to N2a cells in preseeded 96‐well culture dishes followed by 24 h of incubation. After relevant incubations, cells were treated with MTT (10 μL per well) for 4 h, and then DMSO (150 μL per well) was added. Optical density was recorded at 490, 570, and 630 nm.

#### 2.4.2 Flow cytometry analysis of apoptosis

Apoptosis was analyzed by flow cytometry using an annexin V‐FITC/PI apoptosis detection kit (Beyotime Biotechnology, Beijing, China) following the manufacturer's instructions. N2a cells were harvested after being cultivated in six‐well culture dishes under the conditional medium for 24 h. The conditional medium was collected as follows: First, the BV2 cells were plated in six‐well culture plates (6 × 10^5^ cells per well) in the culture medium. Then the BV2 were pretreated with PA for 2 h, and subsequently, incubated with Aβ42 oligomers for 10 h. The supernatant of BV2 cells was replaced with fresh medium for an additional 12 h to be a conditional medium. N2a cells were washed twice with precooled PBS. Staining of the cells was carried out with Annexin V‐FITC plus propidium iodide with protection from light for 10 min. Then the cells were examined and analyzed in FACScan using Cell Quest software (BD Biosciences, USA).

### 2.5 Enzyme‐linked immunosorbent assay

The mice's brains were separated and homogenized in PBS. The homogenates were then centrifuged at 12,000 *g* under 4°C for 15 min, and the supernatant fraction was collected. BV2 cells were plated in six‐well culture plates (6 × 10^5^ cells per well) in the culture medium. BV2 were pretreated with 50 μM PA for 2 h and then stimulated with 5 μM Aβ42 oligomers for 24 h. For the mechanism analysis, BV2 were pretreated with 20 μM GW9662 for 1 h, PA for 2 h, and then treated with 5 μM Aβ42 oligomers for 24 h. The pro‐inflammatory factors including TNF‐α, IL‐1β, and IL‐6 in the hippocampus tissue and BV2 cell supernatants were detected using the ELISA kits based on standard protocol.

### 2.6 Immunofluorescence analysis

The paraffin‐embedded brain tissue was sliced into brain sections (5 μm thickness) using a microtome (LEICA, Germany) for immunofluorescent staining. The sections of hippocampus were conducted with immunochemistry staining of Iba‐1 and NeuN. The sections were incubated with antigen repair solution (0.1%) after dewaxing and hydration in xylene and gradient ethanol solution and blocked with 2.5% blocking goat serum for 1 h at room temperature. The sections were then incubated with primary antibodies overnight at 4°C. Followed by responsive secondary antibodies stained for 2 h and counterstained with 4,6‐diamino‐2‐ phenyl indole (DAPI) for 15 min. BV2 cells were seeded in six‐well culture plates (6 × 10^5^ cells per well) in the culture medium. The cells were pretreated with or without GW9662 for 1 h and PA for 2 h before being incubated with Aβ42 oligomers. After incubation, the cells were fixed in cool paraformaldehyde for 15 min and then treated with 0.3% Triton X‐100 for 20 min. After blocking in goat serum for 30 min, the cells were incubated overnight at 4° with primary anti‐NF‐κB p65 antibody. The cells were incubated with Alexa Fluor goat anti‐mouse IgG for 1 h, and DAPI was used to label the nuclei. The immunofluorescent sections were visualized, and the images were captured using an Olympus fluorescence microscope (Olympus, Japan).

### 2.7 Western blot analyses

Protein extraction reagents were used to extract proteins from mouse brains or cells. The nuclear and cytosolic proteins were extracted and isolated using the Nuclear and Cytoplasmic Protein Extraction Kit (Sangon Biotechnology) according to the manufacturer's instructions. Total protein concentrations were determined with the BCA protein assay kit (Beyotine, Shanghai, China). Protein samples were separated via sodium dodecyl sulphate polyacrylamide gel and transferred onto polyvinylidene fluoride membranes (Millipore, Billerica, MA, USA). The membranes were blocked in 5% skim milk with TBST buffer for 2 h at room temperature, and incubated with specific primary antibodies at 4°C overnight. After washing three times with TBST buffer, the membranes were probed with appropriate corresponding rabbit or mouse secondary antibodies at room temperature for 2 h. Finally, the protein blots were visualized by the chemiluminescence reaction using the enhanced chemiluminescence kit (Millipore, Billerica, MA, USA) and detected by the Bio‐Rad gel imaging system. The relative band intensities were quantified by ImageJ software (Image J version 1.51e, National Institutes of Health, Maryland, USA).

### 2.8 Statistical analysis

All data were statistically analyzed by GraphPad Prism 5.0 (GraphPad Prism Software, CA) and expressed as mean ± standard deviation (SD). For the Morris water maze analysis, repeated measures were analyzed by two‐way analysis of variance (ANOVA). Other differences between the groups were determined with one‐way ANOVA. All experimental results were obtained from at least three independent experiments. The value of *p* < 0.05 was taken as statistically significant.

## 3 RESULTS

### 3.1 PA ameliorates Aβ42 oligomer‐induced learning and memory deficits in mice

**FIGURE 1:**
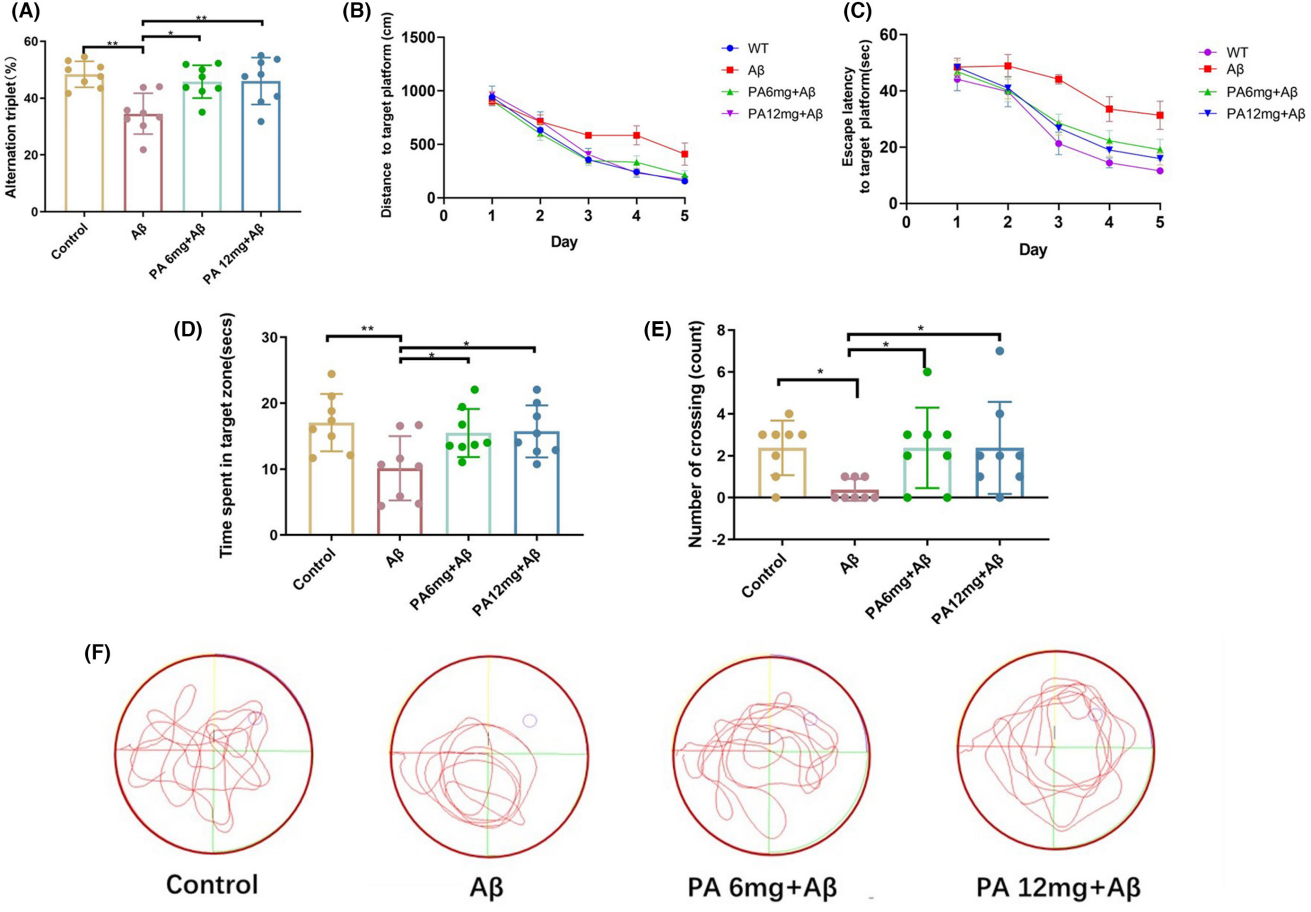
PA ameliorates Aβ42 oligomer‐induced learning and memory deficits in mice. (A) The spontaneous alternation rate in Y‐maze test. (B) Distance and (C) escape latency to the target platform on the learning phase of the Morris water maze assessment. (D) Time spent on the target quadrant during the spatial probe phase. (E) A number of cross the platform during the spatial probe trial. (F) Representative tracing recordings for the spatial probe trial (each group *n* = 8). *p < 0.05, **p < 0.01, ***p < 0.001; distance traveled to target was analyzed using two‐way analysis of variance (ANOVA), and the others using one‐way ANOVA with Tukey's post‐hoc test. Bars represent mean ± SD.

The results of Y‐maze showed that the spontaneous alternation rates of Aβ42‐injected AD model group were lower than the control group, while PA treatment groups showed higher alternation rates than those of the AD model group, suggesting that mice treated with PA improved the spatial memory impairment induced by Aβ42 (Figure 1A). Furthermore, the results of Morris water maze showed that AD model mice had less reduction in the distance and escape latency to the target during the learning phase. However, PA‐treated groups showed significant decrease on the distance (Figure 1B) and escape latency (Figure 1C) over the course of the learning phase, indicating that compared with AD model mice, the treatment of PA significantly improved the learning speed of mice. In the spatial probe trial phase, AD model mice spent less time on the target quadrant and showed fewer platform site crossings than the control group. The PA treatment groups showed more time in the target quadrant (Figure 1D) and more crossing numbers (Figure 1E) than the AD model mice group, demonstrating that the PA treatment ameliorated the cognitive impairment induced by Aβ42. The representative figure on the spatial probe phase is shown in Figure 1F.

### 3.2 PA inhibits inflammation response induced by Aβ42 oligomer in mice

**FIGURE 2:**
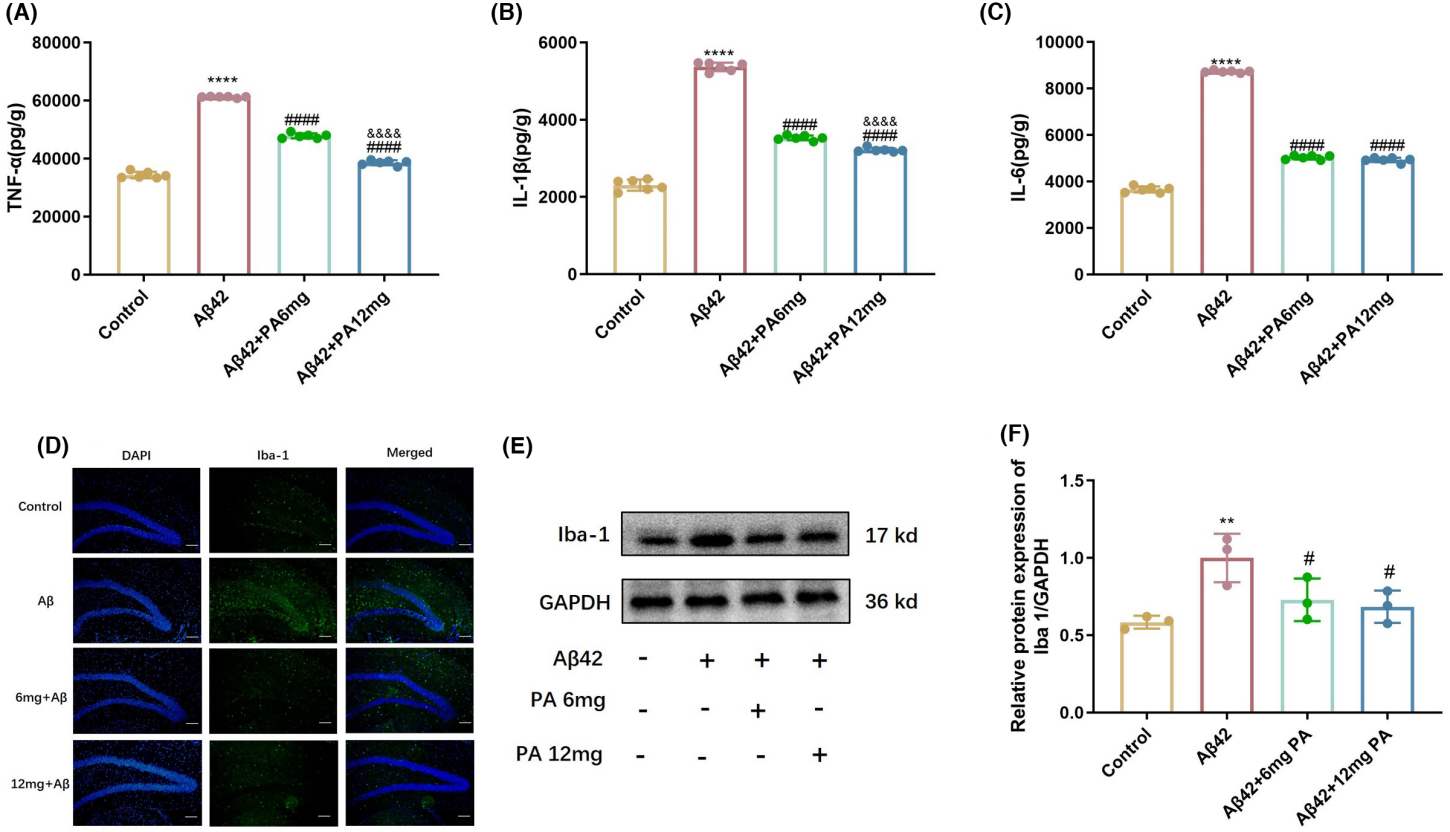
Inhibitory effects of PA on inflammation induced by Aβ42 oligomer in mice. (A) TNF‐α, (B) IL‐1β, and (C) IL‐6 were examined using ELISA (*n* = 6 per group). (D) Immunofluorescence analysis of Iba‐1, bar = 25 μm. (E) Western blots and (F) quantitative analysis of Iba‐1 (*n* = 3 per group). All values are presented as the mean ± SD. *p < 0.05, **p < 0.01, and ***p < 0.001 compared with the control group. ^#^ *p* < 0.05, ^##^ *p* < 0.01, ^###^ *p* < 0.001, and ^####^ *p* < 0.001 compared with the AD model group.

ELISA results showed that the expression of pro‐inflammation factors in the brain tissue of mice was increased in the AD model group. However, PA treatment inhibited the expression of pro‐inflammation factors. The results showed that TNF‐α decreased by 22% in the 6 mg/kg PA treatment group and by 37% in the 12 mg/kg PA treatment group (Figure 2A). Results for IL‐1β were similar to TNF‐α. The expression of IL‐1β decreased by 36% in 6 mg/kg PA treatment group and by 42% in 12 mg/kg PA treatment group (Figure 2B). As for IL‐6, the inflammation factor IL‐6 in the 6 mg/kg dose group decreased by 43% compared with that in the AD model mice, and in the 12 mg/kg dose group, it decreased by 44% (Figure 2C).

Ionized calcium‐binding adapter molecule‐1 (Iba‐1) plays a critical role in the regulation of the immunological function of microglia and acts as a unique marker for microglial. Immunofluorescence analysis showed an increased number of Iba‐1‐positive cells in the hippocampus of AD model mice, while the number of Iba1‐positive cells was reduced in the PA treatment groups (Figure 2D). Western blot results showed a high expression of Iba‐1 in the brains of AD model mice and a significantly decreased expression in the PA treatment groups (Figure 2E,F). These results suggest that PA could inhibit the inflammation response induced by Aβ42 oligomer in mice.

### 3.3 Network pharmacology and molecular docking showed the potential targets of PA

Network pharmacology and molecular docking were used for target prediction and preliminary validation. Using the SwissTargetPrediction and the SEA platform, 62 and 19 potential targets of PA were obtained, respectively. By merging the results and removing duplicated targets, a total of 73 targets of PA were identified, as shown in Figure S1A. Through GeneCards and OMIM databases, AD targets were retrieved and collected. A total of 11,716 AD‐related targets were obtained when the duplicated and summarized results. After the intersection, 60 common targets between AD and PA were obtained and then used for the PPI analysis. The PPI results are shown in Figure S1B, which suggested that PTGS2 and PPARG were key proteins of PA. PTGS2 is a critical downstream protein of NF‐κB and was mainly involved in inflammation.

The GO functional enrichment showed 30 BP items, 19 cellular components (CC), and 50 MF items with statistical significance (*p* < 0.05). Figure S2A displays the top 10 BP, CC, and MF items, which were mainly associated with inflammation response, response to lipopolysaccharide, and positive regulation of gene expression or cell proliferation, as illustrated in Figure S2B. Furthermore, the KEGG pathways were displayed based on P‐ranking in Figure S2C, and 18 significant intersection targets were involved. The enrichment results indicated that PPAR‐signaling pathway was one of the common pathways. The molecular docking results showed that PA was likely to bind to Peroxisome proliferator‐activated receptor gamma (PPARγ), forming hydrogen bonds with Arg234 of PPARγ, and the binding energy of PPARγ‐PA was −7.1 kcal/mol (Figure S3).

### 3.4 PA treatment promoted cell viability of Aβ42‐stimulated BV2 microglia and reduced apoptosis of N2a cells

**FIGURE 3:**
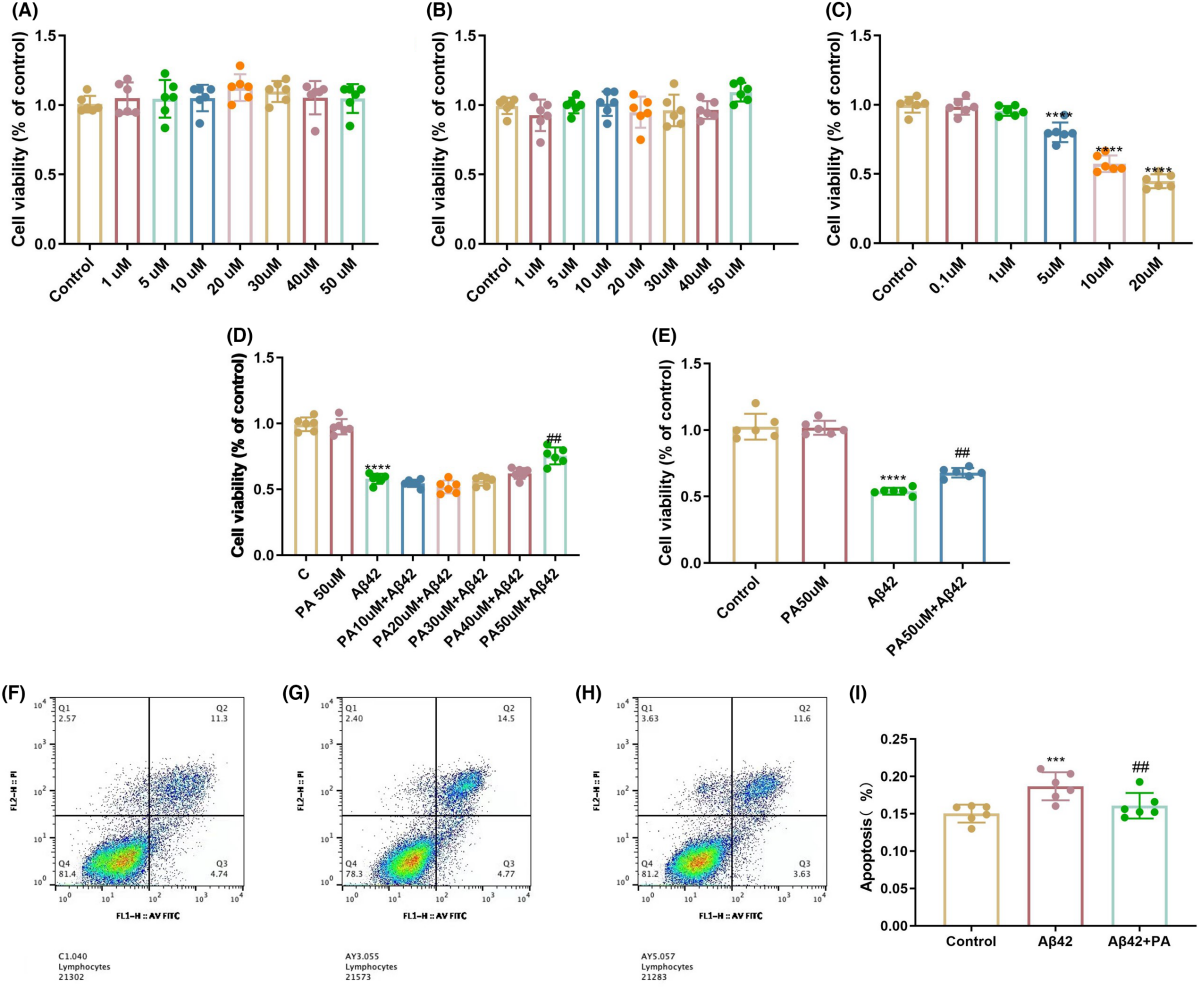
Effect of PA and Aβ42 treatment on the cell viability of BV2 and N2a cells. (A) Cell viability of BV2 cells and (B) N2a cells after 24 h treatment of PA. (C) Cell viability of BV2 cells after 24 h treatment of Aβ42. (D) Cell viability of BV2 cells after 2 h pretreatment with PA followed by 24 h treatment with PA and Aβ42. (E) Cell viability of N2a cells after cultured under conditional medium from BV2. (F–I) Apoptosis of N2a was analyzed by flow cytometry under conditional medium from BV2. *p < 0.05, **p < 0.01, ***p < 0.001, and ****p < 0.0001 compared with the control group. ^#^ *p* < 0.05, ^##^ *p* < 0.01, ^###^ *p* < 0.001, and ^####^ *p* < 0.001 compared with the Aβ42 stimulated group. One‐way ANOVA with Tukey's post‐hoc test. Bars represent mean ± SD.

Cell viability analysis using the MTT assay showed that PA exhibited no toxicity toward BV2 and N2a cells when treated with concentrations of 1, 5, 10, 20, 30, 40, and 50 μM (Figure 3A,B). A concentration of 5 μM Aβ42 can produce significant cytotoxicity to the BV2 cells (Figure 3C). However, treatment with 50 μM PA reversed 5 μM Aβ42 induced cytotoxicity of BV2 (Figure 3D). Further investigation on whether PA could protect N2a cells from Aβ42 oligomer‐induced neurotoxicity. Treatment of N2a cells with a conditioned medium containing Aβ42 significantly decreased N2a cell viability, while 50 μM PA‐pretreated conditioned medium significantly improved the cell viability, of N2a (Figure 3E). Apoptosis analysis by flow cytometry analysis showed that treatment with 50 μM PA could significantly decrease the apoptosis rate of N2a neurons induced by Aβ42 under a conditional medium (Figure 3F–I). Concentrations of 5 μM Aβ42 and 50 μM PA were used for the subsequent experiments.

### 3.5 PA inhibited the neuroinflammation of mice that may be related to PPAR γ/NF‐κB pathway

**FIGURE 4:**
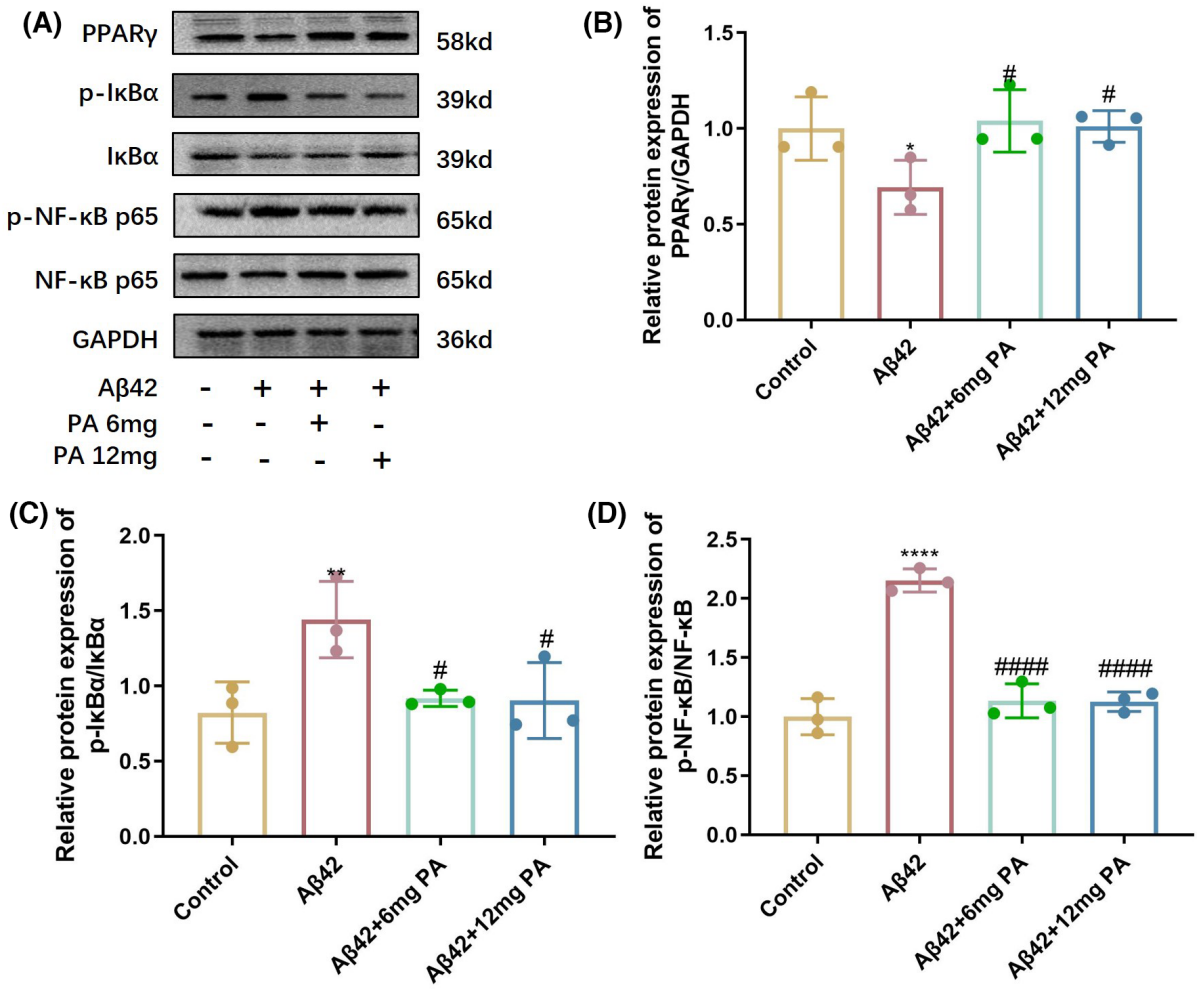
Western blot analysis for PPAR γ/NF‐κB pathway‐related proteins in mice. (A) Western blot results. (B–D) Quantitative analysis of corresponding proteins (*n* = 3 per group). *p < 0.05, **p < 0.01, ***p < 0.001, and ****p < 0.0001 compared with the control group. ^#^ *p* < 0.05, ^##^ *p* < 0.01, ^###^ *p* < 0.001, and ^####^ *p* < 0.001 compared with the Aβ42‐stimulated group. There were eight mice in each group; one‐way ANOVA with Tukey's post‐hoc test. Bars represent mean ± SD.

Based on the results of network pharmalogical analysis, the Western blot analysis was performed to detect the changes in inflammatory signaling pathway‐related proteins in mice. Compared with the control group, the AD model group significantly reduced the expression of PPARγ; the PA treatment increased the expression of PPARγ compared with the AD model group (Figure 4A,B). The expression of phosphorylated NF‐κB/NF‐κB and phosphorylated IκBα was increased in the AD model group, suggesting the activation of the NF‐κB pathway. After PA treatment, compared with the AD model group, the expression of phosphorylated NF‐κB/NF‐κB and phosphorylated IκBα/IκBα was significantly decreased, suggesting the inhibition of the NF‐κB pathway (Figure 4C,D).

### 3.6 PPARγ antagonists reversed the effects of PA on the PPARγ/NF‐κB pathway and downstream inflammation factors in BV2 microglia

**FIGURE 5:**
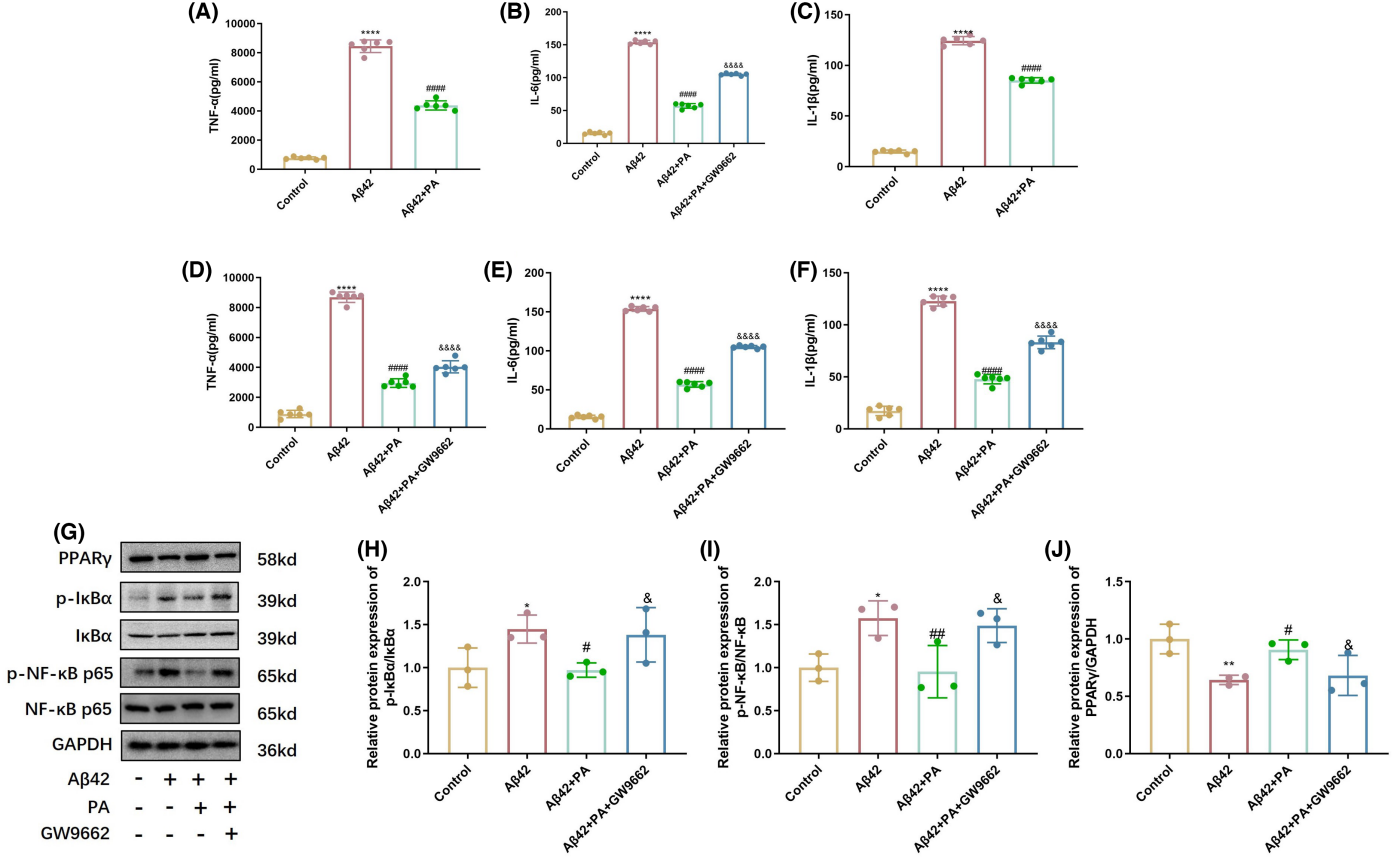
PPARγ antagonists reversed the effects of PA on the PPARγ/NF‐κB pathway and inflammation factors in BV2 microglia. (A–C) The secretion of inflammatory cytokines in BV2 cells after the treatment of PA; (D–F) The secretion of inflammatory cytokines in BV2 cells and after pretreatment with PPARγ inhibitors. (G) Western blot analysis of the PPARγ/NF‐κB pathway and (H–J) quantitative analysis of corresponding proteins. *p < 0.05, **p < 0.01, ***p < 0.001 compared with the control group; ^#^ *p* < 0.05, ^##^ *p* < 0.01, ^###^ *p* < 0.001, and ^####^ *p* < 0.001 compared with the Aβ42 stimulated group; ^&^ *p* < 0.05, ^&&^ *p* < 0.01, ^&&&^ *p* < 0.001, and ^&&&&^ *p* < 0.001 compared with the PA treatment group. One‐way ANOVA with Tukey's post‐hoc test. *n* = 6 per group for ELISA analysis, and *n* = 3 for Western analysis. Bars represent mean ± SD.

The BV2 microglia pretreated with PA for 2 h prior to Aβ42 stimulation showed a lower level of TNF‐α, IL‐6, and IL‐1β than the Aβ42‐stimulated group (Figure 5A–C). After adding the PPARγ‐specific inhibitor, GW9662, before the PA treatment, the results showed an increased level of inflammation factors, suggesting the protective effect of PA was significantly reduced by the PPARγ inhibitor (Figure 5D–F).

The Western blot analysis showed that compared with the Aβ42 oligomer stimulation group, pretreatment with PA significantly increased the expression of PPARγ protein and decreased the ratio of p‐NF‐κB (p65)/NF‐κB and p‐IκBα/IκBα. Nevertheless, after adding GW9662 before pretreatment with PA, the effects of PA on PPARγ/NF‐κB pathway proteins were weakened. Compared with the pretreatment with PA group, the expression of PPARγ protein was decreased, and the ratio of p‐NF‐κB (p65)/NF‐κB and p‐IκBα/IκBα was relatively increased in the inhibitor group (Figure 5G–J, *n* = 3 per group).

### 3.7 The effect of PA on NF‐κB nuclear translocation was suppressed by PPARγ antagonists in Aβ‐activated BV2 microglia

**FIGURE 6:**
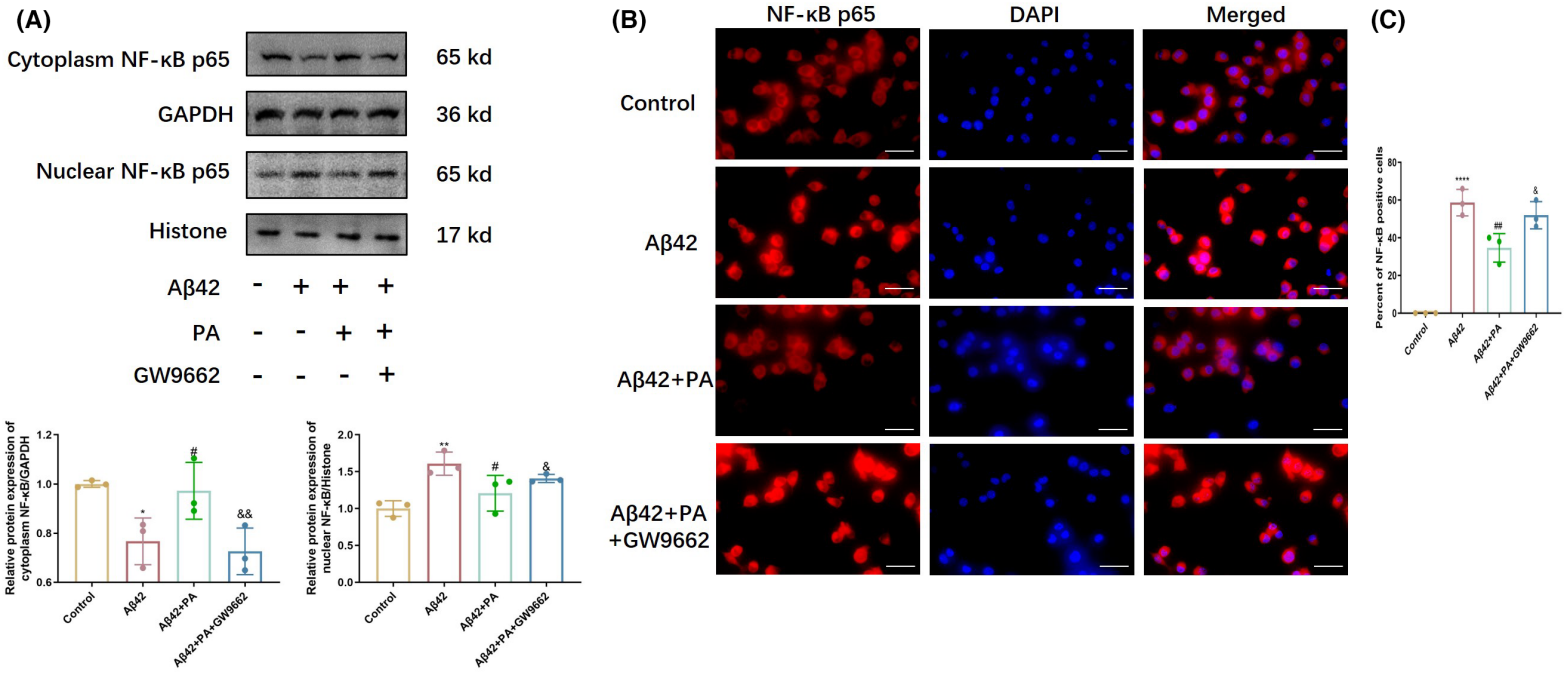
NF‐κB expressions in the nucleus and cytoplasm after adding the inhibitor of PPARγ. (A) Western blots analysis for NF‐κB p65 subunit in nuclear and cytosolic and quantitative analysis of corresponding proteins. (B) The representative immunofluorescence images showing translocation of NF‐κB (p65, red) to the nucleus (blue) after treatment with Aβ42 oligomers or pretreat with PA or adding the inhibitor of GW9662. Bar = 50 μm. (C) Percent of nuclear NF‐κB p65‐positive cells. Values were expressed as the mean of at least three independent replicates, whose means resulted from 50 cells from three independent fields of view. *p < 0.05, **p < 0.01, ***p < 0.001, ****p < 0.0001 compared with control group; ^#^ *p* < 0.05, ^##^ *p* < 0.01, ^###^ *p* < 0.001, and ^####^ *p* < 0.001 compared with the Aβ42 stimulated group; ^&^ *p* < 0.05, ^&&^ *p* < 0.01, ^&&&^ *p* < 0.001, and ^&&&&^ *p* < 0.001 compared with the PA treatment group. *n* = 3 for Western analysis, one‐way ANOVA with Tukey's post‐hoc test. Bars represent mean ± SD.

To further investigate the effect of PA on the PPARγ/NF‐κB pathway, we detected the expression of NF‐κB (p65) protein in the cytoplasm and nucleus, respectively. Aβ42 oligomers decreased the expression of NF‐κB (p65) in the cytoplasm significantly, while increasing that in the nucleus. PA treatment resulted in opposite results to the Aβ42 oligomer stimulation group, with significantly increased expression of NF‐κB (p65) protein in the cytoplasm and decreased in the nucleus. However, after treatment with the PPARγ inhibitor, the effects of PA on the expression of NF‐κB (p65) protein in the cytoplasm and nucleus were suppressed, and more NF‐κB (p65) expressed in the nucleus (Figure 6A). Immunofluorescence staining also showed that GW9662 increased the transfer of NF‐κB (p65) protein into the nucleus, indicating the effect of PA on NF‐κB nuclear translocation was PPARγ‐dependent (Figure 6B,C).

## 4 DISCUSSION

In the present study, we assessed the anti‐inflammatory effect of PA and its underlying mechanisms based on overproduction network pharmacology and molecular docking. We found that PA improved cognitive impairment, reduced the over production of proinflammatory cytokines, increased the expression of PPARγ, and inhibited the activation of the NF‐κB signaling pathway in AD model mice and Aβ‐stimulated BV2 microglia. Furthermore, PA could also reduce the apoptosis of N2a neurons via attenuated BV2 microglia‐mediated inflammation induced by Aβ42 oligomers. As far as we know, this is the first study to demonstrate that PA can decrease the proinflammatory cytokines in the AD model mice and BV2 microglial cells through the PPARγ/NF‐κB pathway.

Inflammation is the prime factor involved in the progression of various diseases including neurodegenerative diseases, which is validated by the increase in the expression of proinflammatory cytokines both in the brain tissues and the blood samples of AD patients and AD mice model.^28^, ^29^ Proinflammatory cytokines are the key players in the neuroinflammation process and can be produced by microglial cells. The aggregation and accumulation of the Aβ protein excessively stimulate microglia, causing them to become overactive. Activated microglia cells participated in neuroinflammatory events directly via proinflammatory mediators' production such as IL‐1β, IL‐6, and TNF‐α.^30^ TNF‐α is considered to be the most critical proinflammatory factor in AD, which promotes and modulates the cytokine members during the process of inflammation.^31^ Studies suggested that overexpression of TNF‐α in triple‐transgenic AD mice leads to an increase in intracellular Aβ, enhanced inflammation, and neuronal cell death.^32^ In contrast, inhibition of TNF‐α production may protect neurons from death.^33^ IL‐1β, a member of the IL‐1 cytokine family, is considered a major pro‐inflammatory cytokine and plays a critical role in the progression of AD. Increased serum levels of IL‐1β are used as a stage marker of the ongoing brain neurodegeneration in the continuum between normal aging and AD.^34^ IL‐1β production is associated with synaptic plasticity and is also involved in the formation of NFT.^35^ The expression of IL‐6 is significantly increased around amyloid plaques in AD patients and animal models. The IL‐6 gene corresponds to chromosome p21 and is a promising candidate for a genetic risk factor for AD.^36^ The IL‐6 gene polymorphisms are related to AD risk.^37^ IL‐6 stimulates and increases the migration of microglia and astrocytes in the AD brain, causing them to produce pro‐inflammatory cytokines and promoting tau phosphorylation in neurons.^38^ Simultaneously, these pro‐inflammatory chemicals could damage healthy neurons, and as more neurons become damaged, this further activates microglia, thus increasing the production of inflammatory mediators and eliciting neurodegeneration. Therefore, inhibiting Aβ‐induced microglial activation can reduce the production of TNF‐α, IL‐1β, and IL‐6, thereby reducing Aβ deposition and neuron death, blocking the cycle of inflammatory toxicity, and improving behavioral performance in AD mice. In this study, the proinflammatory factors were increased both in the Aβ‐stimulated BV2 microglia and AD mice model. However, the PA treatment could significantly inhibit the overproduction of proinflammatory cytokines IL‐1β, IL‐6, and TNF‐α in vivo and in vitro. Thus, our results indicated that PA has the ability to attenuate neuroinflammation by inhibiting the activation of microglial cells and overproduction of proinflammatory mediators.

Furthermore, we explored the mechanisms of PA attenuating the inflammation based on the network pharmacology. The results suggested that PPARγ was the key protein of PA and PPAR‐mediated signals pathway was one of the main pathways. The molecular docking results suggested PA could bind the PPARγ tightly. PPARγ was a ligand‐activated nuclear transcription factors and was reported to be involved in repressing the expression of inflammation‐related genes.^39^, ^40^ Activation of PPARγ and subsequent regulation of gene transcription requires binding of ligand. Previous studies showed that activation of PPARγ improved both learning and memory along with other AD‐related pathology.^41^, ^42^, ^43^ Transgenic mouse model of AD shows that Pioglitazone, a PPARγ agonist, reduced activation of microglial and reduction in soluble and insoluble Aβ levels.^44^ In addition, injection of PPARγ agonist rosiglitazone into the brain of Aβ oligomers treated rats prevented the increase of inflammatory cytokines levels, and this is related to improvement in cognitive decline and prevention of microglia activation.^45^ Our study showed that PA could up‐regulate the expression of PPARγ in AD model mice and Aβ‐stimulated BV2 microglia, suggesting PA might inhibit the inflammation through binding PPARγ, and supporting that PA might be a potential agonist of PPARγ.

Further studies have shown that PPARγ can suppress pro‐inflammatory gene expression by reducing NF‐κB transcriptional activity or by interacting with activated p65.^46^ These results suggested the NF‐κB pathway might be involved in the effects of PPARγ modulators.^47^, ^48^ NF‐κB plays a critical role in neurodegenerative diseases by regulating microglia‐mediated neuroinflammation.^49^ IκBα mediated nuclear export of the NF‐κB complex appears to be the central mechanism terminating NF‐κB signaling. In inactive cells cytoplasm, NF‐κB with p65–p50 dimer interacts with IκBα to form inactive complexes. After stimulation by Aβ42 or proinflammatory factors, IκBa is immediately phosphorylated and further degraded, the released NF‐κB dimers are then activated and translocate to the nucleus to regulate the expressions of target genes.^50^ Activation of the NF‐κB signaling pathway promotes the release of cytokines and chemokines from microglia, leading to the chronic inflammatory response observed in AD. In our current study, we found up‐regulation of PPARγ expression significantly inhibited Aβ42 oligomer‐induced activation of the NF‐κB signaling pathway, which reversed the antagonization of PPARγ with GW9662. Consistent with our results, the previous study also found activation of PPARγ could repress the expression of inflammatory factors by negatively interfering with the NF‐κB pathway.^39^ This suggested PA might inhibit the inflammation through the PPARγ/NF‐κB pathway in treating AD.^51^ We also explored the downstream effect of activation of the PPARγ/NF‐κB pathway after PA treatment. The results showed the PA treatment decreased apoptosis in conditioned medium‐cultured N2a cells. These results were consistent with previous studies, which suggested that ligand‐activated PPARγ induced cell apoptosis by blocking the anti‐apoptotic signaling of NF‐κB.^52^

The strength of this study is that it demonstrates for the first time the inflammation and apoptosis inhibition by PA in AD models and proves its effectiveness in both neurons and microglia. There are also some limitations in this study. First, we adopted the Aβ42‐injected model to verify the role of PA in AD, and further validation in transgenic AD mice is needed. Second, PA may exert neuroprotective effects on AD through a multi‐target and multi‐pathway approach based on the results of network pharmacology. However, this study mainly explores the anti‐inflammatory effects of PA and its mechanisms. Further studies are needed to explore the multi‐target characteristics of PA.

## 5 CONCLUSIONS

In conclusion, we elucidated that PA can improve cognitive function by inhibiting inflammation and reducing apoptosis. This effect was mediated by the activation of the PPARγ/NF‐κB signaling pathway. These findings suggest anti‐inflammation as a potential target to treat AD and PA could be a potential therapeutic agent for the development of AD treatment.

## FUNDING INFORMATION

This study was supported by the Innovation Center for Neurological Disorders and the Department of Neurology at Xuanwu Hospital. This study was supported by the Key Project of the National Natural Science Foundation of China (81530036), the National Key Scientific Instrument and Equipment Development Project (31627803), Beijing Scholars Program, Beijing Brain Initiative from Beijing Municipal Science and Technology Commission (Z201100005520016 and Z201100005520017), and the Key Project of the National Natural Science Foundation of China (U20A20354).

## CONFLICT OF INTEREST STATEMENT

The authors confirm that they have no conflict of interest.

## CONSENT

The authors have read the manuscript and agreed to publish.

## Supporting information


Figures S1‐S3.

## Data Availability

All data analyzed and presented in this study are available from the corresponding author upon reasonable request.
