# Supplementary material for: Polygalacic acid attenuates cognitive impairment by regulating inflammation through PPARγ/NF‐κB signaling pathway
Source: CNS Neurosci Ther. 2024 Feb 8;30(2):e14581. doi: 10.1111/cns.14581 (PMC10851321; doi:10.1111/cns.14581)
Supplement: Supplementary file 1 — Figures S1‐S3. [file CNS-30-e14581-s001.docx]

Supplementary


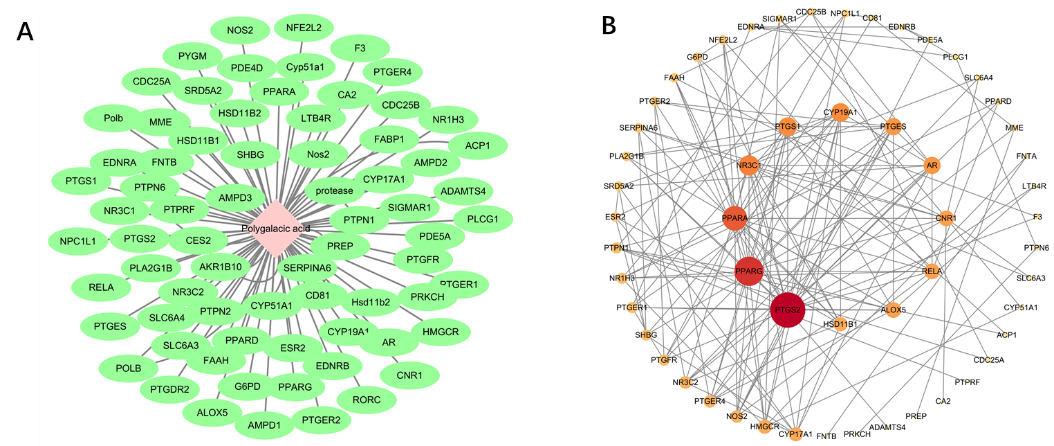


Figure 1. Network pharmacology showed the potential targets of PA. (A) The potential target of PA. (B) PPI network of targets.


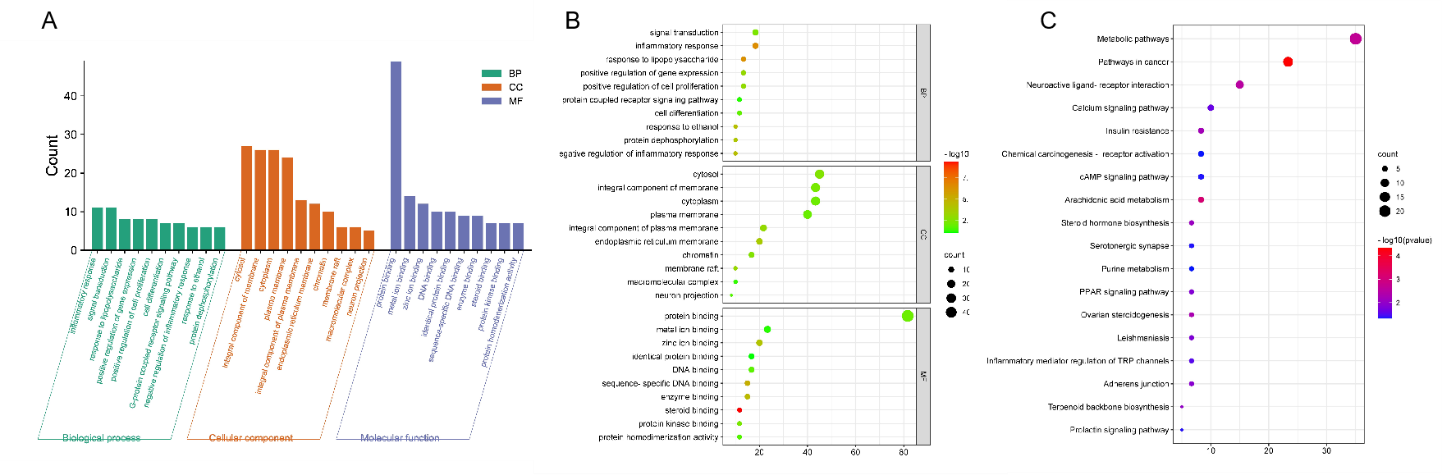


Figure 2. The GO enrichment and KEGG analysis. (A-B) GO Enrichment. (C) KEGG Enrichment.


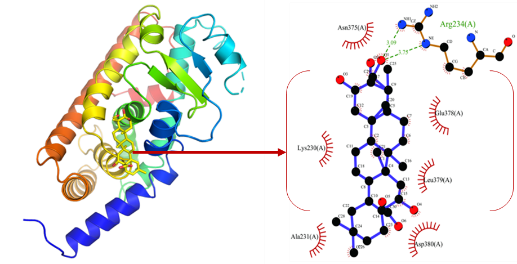


Figure 3. Molecular docking results of PPARγ and PA.
